# Copper‐Based Nanotubes That Enhance Starvation Therapy Through Cuproptosis for Synergistic Cancer Treatment

**DOI:** 10.1002/advs.202504121

**Published:** 2025-06-05

**Authors:** Xuan Han, Xin Zhang, Zhiping Liu, Hang Liu, Derek Wu, Yinli He, Kexin Yuan, Yi Lyu, Xiaofei Liu

**Affiliations:** ^1^ Department of Hepatobiliary Surgery The First Affiliated Hospital of Xi'an Jiaotong University Xi'an Shaanxi Province 710061 China; ^2^ Center for Regenerative and Reconstructive Medicine Med‐X Institute The First Affiliated Hospital of Xi'an Jiaotong University Xi'an Shaanxi Province 710061 China; ^3^ Department of Orthopedics The Second Affiliated hospital of Air Force Medical University (AFMU) Xi'an Shaanxi Province 710000 China; ^4^ National Local Joint Engineering Research Center for Precision Surgery & Regenerative Medicine Shaanxi Provincial Center for Regenerative Medicine and Surgical Engineering The First Affiliated Hospital of Xi'an Jiaotong University Xi'an Shaanxi Province 710061 China; ^5^ Brooklyn College the City University of New York Brooklyn NY 11210 USA; ^6^ BioBank The First Affiliated Hospital of Xi'an Jiaotong University Xi'an Shaanxi Province 710061 China

**Keywords:** cuproptosis, hepatocellular carcinoma, starvation therapy

## Abstract

Hepatic cancer is the leading cause of worldwide death due to lack of effective chemotherapy drugs. Cuproptosis is a novel regulated cell death and offers a new potential mechanism to eliminate hepatic cancer. In this study, it is found that hepatocellular carcinoma (HCC), which is one of the hepatic cancer, is sensitive to cuproptosis by analysing clinical specimens and databases. Thus, a copper‐based nanotube (NT) with copper ionophores to induce enhanced cuproptosis is designed. In combination with starvation therapy, the NT significantly inhibited energy metabolism in HCC cells. This results show that the depletion of GSH by Cu^2+^ intensified oxidative stress and cuproptosis, resulting in ROS generation and DLAT aggregation, indicating the enhancement of cuproptosis and the Fenton reaction. The NT with synergetic cuproptosis, the Fenton reaction and starvation therapy provide a valuable insight into the prospect of HCC treatment.

## Graphical abstract

We designed a copper‐based nanotube (NT) with copper ionophores to induce enhanced cuproptosis. In combination with starvation therapy, the NT significantly inhibited energy metabolism in HCC cells. Our results show that the depletion of GSH by Cu^2+^ intensified oxidative stress and cuproptosis, resulting in ROS generation and DLAT aggregation, indicating the enhancement of cuproptosis and the Fenton reaction. The NT with synergetic cuproptosis, the Fenton reaction and starvation therapy provide a valuable insight into the prospect of HCC treatment.

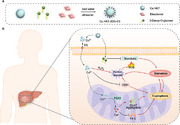

## 1 Introduction

Hepatic cancer, which primarily consists of hepatocellular carcinoma (HCC), seriously jeopardizes human health and caused 830180 deaths in 2020.^[^ ^1^ ^]^ However, most HCC cells are not sensitive to chemotherapy, resulting in a dilemma for patients with advanced HCC.^[^ ^2^, ^3^ ^]^

Cuproptosis, a recently identified form of regulated cell death, is expected to be a novel therapeutic strategy for HCC because the liver plays a central role in copper metabolism and imbalances in copper metabolism can adversely affect the liver. It is triggered by excessive accumulation of intracellular copper, which leads to the aggregation of mitochondrial protein and the subsequent loss of Fe‐S cluster protein, leading to tricarboxylic acid (TCA) cycle disruption and cell death.^[^ ^4^ ^]^ But currently the studies are mainly focused on genomics research, exploring the potential use of genes related to cuproptosis in the treatment of HCC.^[^ ^5^ ^]^ The fundamental research between HCC and cuproptosis was merely investigated and needed to be filled. Meanwhile, several copper ionophores were developed in the past decades for tumor treatment.^[^ ^6^, ^7^ ^]^ For example, elesclomol (ES) presented substantial effectiveness in cancer patients with high lactic levels.^[^ ^8^ ^]^ Nevertheless, the effect of cuproptosis was limited in glycolysis‐dependant tumor cells, and copper ionophores need to be enhanced to induce a satisfied outcome.^[^ ^8^ ^]^ And nanomedicine offers new insights into the development of cuproptosis‐based drugs for tumor therapy.

In recent years, the mechanism of ROS in tumorigenesis, which including hydrogen peroxide (H_2_O_2_), singlet oxygen (^1^O_2_) and free oxygen radicals in various forms,^[^ ^9^, ^10^ ^]^ and development has been widely studied and explored, confirming that in tumor cells there is an over‐accumulation of ROS due to the dysregulation of ROS production‐elimination balance.^[^ ^11^, ^12^ ^]^ Then Cu ion is discovered to catalyze the overexpressed H_2_O_2_ to toxic •OH in tumor tissue by the Fenton reaction.^[^ ^12^, ^13^ ^]^ Moreover, copper ions can also catalyze the oxidation of glutathione (GSH), prompting the conversion of GSH to glutathione disulfide (GSSG), which can increase the sensitivity of cells to cuproptosis.^[1^ ^4^ ^]^

Therefore, elevating the concentration of Cu ion and the levels of ROS can effectively treat HCC due to their synergistic effect.

At the same time, the tumor cells consume much more glucose for glycolysis than normal cells.^[^ ^15^ ^]^ Therefore, blocking tumor cells from glucose becomes a promising strategy to disturb cellular metabolism and to induce cell death, prompting much research on glucose analog or oxidase.^[^ ^16^ ^]^ 2‐deoxyglucose (2DG) is used as a glucose analog for starvation therapy, which can reduce tumor cells' energy supply, limit their proliferation, and aggravate the redox balance owing to a decrease in reducing agents.^[^ ^17^ ^]^ Nevertheless, tumor cells can alleviate the effect of 2DG by utilizing the TCA cycle. Thus, inhibiting glycolysis and the TCA cycle is vital for tumor starvation therapy.^[^ ^18^ ^]^

**Scheme 1:**
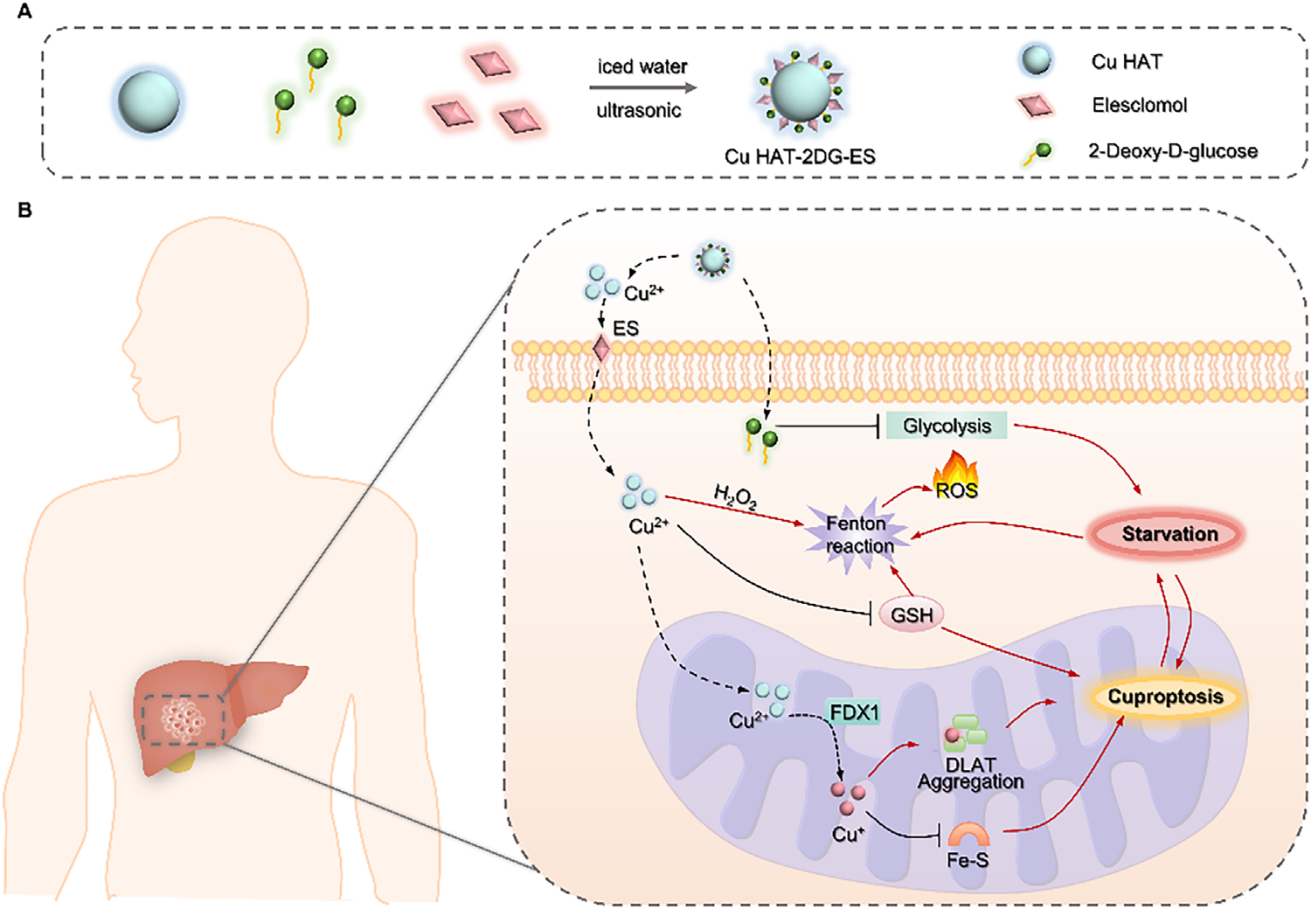
Schematic of the Cu HAT‐2DG‐ES for enhanced CDT and starvation therapy with cuproptosis against HCC. A) Design of Cu HAT‐2DG‐ES. B) Cu HAT‐2DG‐ES induces synergistic CDT and starvation therapy with cuproptosis.

In conclusion, Copper‐based nanomaterials can provide sufficient Cu ion to induce effective cuproptosis, thereby inhibiting the TCA cycle, and serve as delivery platforms with the Fenton reaction and starvation therapy, which is a potential therapeutic measure for HCC. Thus, we designed a copper‐based nanoplatform for enhanced cuproptosis, the Fenton reaction and starvation therapy for HCC treatment. In this nanotube (NT), copper‐based crystal Cu HAT was synthesized and loaded with ES and 2DG, which catalyzed the Fenton reaction to generate excess ROS in cells. The released copper ions could be efficiently transported into the cells by ES to induce cuproptosis. At the same time, Cu ion can oxidize intracellular GSH, enhancing cuproptosis. 2DG and cuproptosis synchronously inhibit glycolysis and TCA cycling in tumor cells, synergistically disrupting the energy metabolism of tumor cells, worsening the redox imbalance, and enhancing the effects of the Fenton reaction and cuproptosis. The NT developed based on this background can effectively induce cuproptosis in hepatocellular carcinoma and inhibit the growth of hepatocellular carcinoma in mice (**Scheme** **1**).

## 2 Results and Discussion

### 2.1 Potential Validity of Cuproptosis‐Based Treatment for HCC

**Figure 1:**
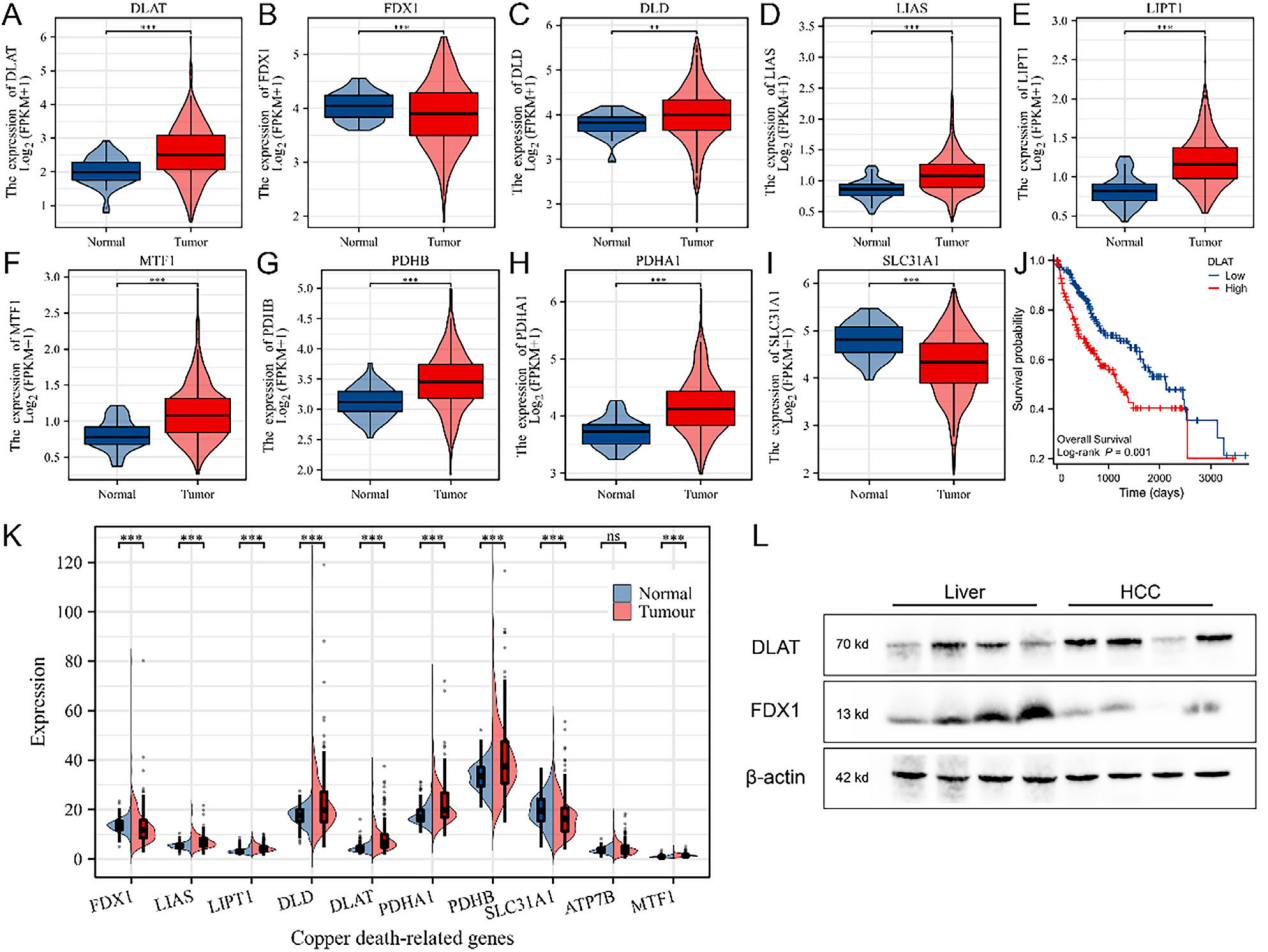
A–I) Cuproptosis‐related genes expression in TCGA database. J) Survival analysis of DLAT. K) Cuproptosis‐related gene expression in the ICGC database. L) DLAT and FDX1 expression in HCC and normal tissuespecimen.

We investigated cuproptosis‐related protein expressions in human HCC specimens and ICGC and TCGA databases. As shown in **Figures** **1**A–I, K and S2A–Q (Supporting Information), cuproptosis‐related gene expression in HCC significantly differs from normal tissue. In particular, DLAT, one of the most critical targets of cuproptosis, has been overexpressed in HCC and highly correlated with survival (Figure 1J,L; Figure S2R, Supporting Information), indicating the potential validity of cuproptosis‐based treatment for HCC.

### 2.2 Synthesis and Characterization

**Figure 2:**
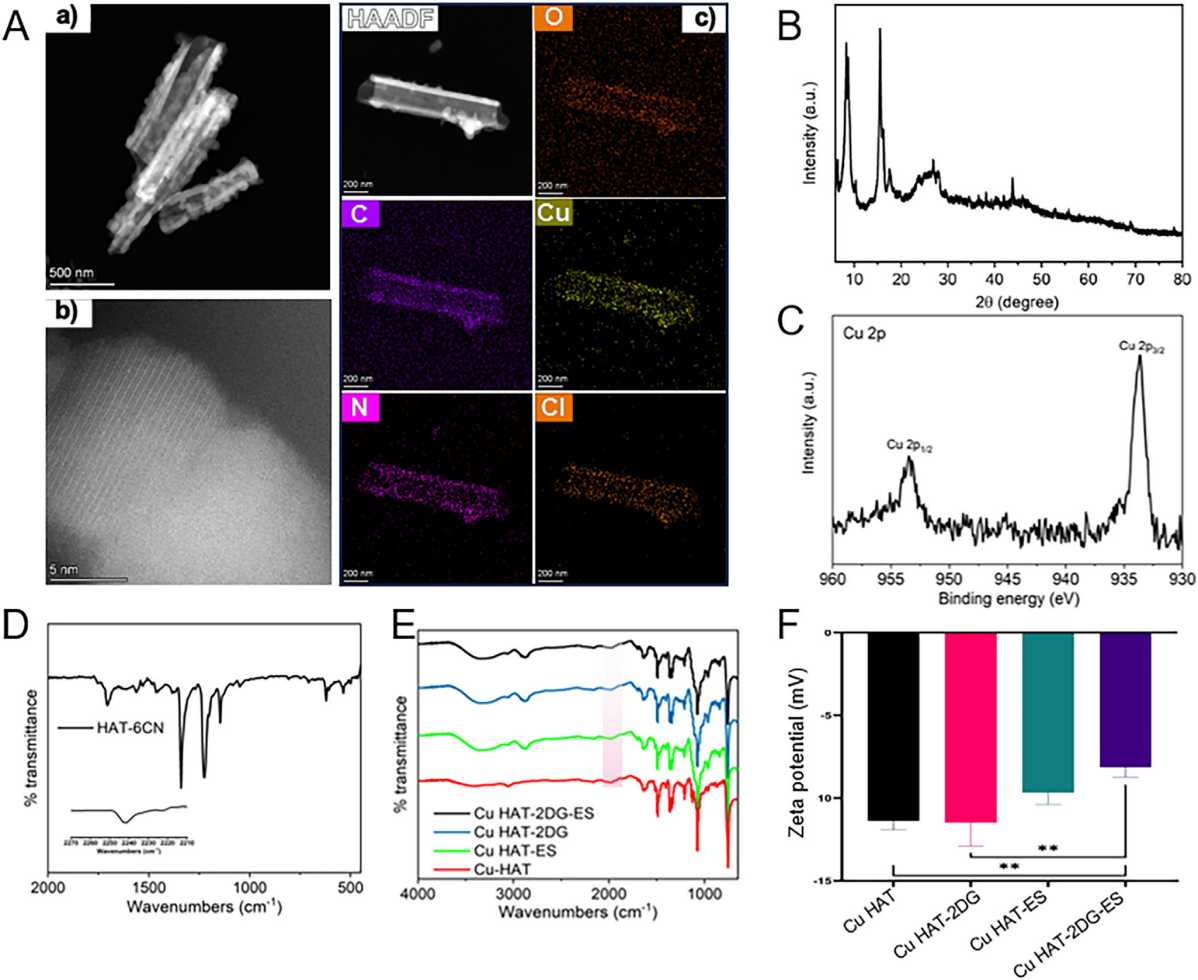
A) High‐resolution TEM images (a,b) and elemental mapping (c) of Cu‐HAT‐6CN, respectively. B) XRD pattern of Cu‐HAT‐6CN. C) High‐resolution XPS spectrum of Cu 2p for Cu‐HAT‐6CN. D) FTIR pattern of HAT‐6CN. E) FTIR pattern of Cu HAT, Cu HAT‐2DG, Cu HAT‐ES and Cu HAT‐2DG‐ES, respectively. F) Zeta potentials of Cu HAT, Cu HAT‐2DG, Cu HAT‐ES and Cu HAT‐2DG‐ES, respectively. (*n* = 5, *** indicates p < 0.001, ** indicates p < 0.01, * indicates p < 0.05).

The Cu HAT‐6CN complex was synthesized through a straightforward air evaporation process involving a solution comprising Cu(ClO_4_)_2_ dissolved in water and HAT‐6CN dissolved in acetonitrile. A nanotube was successfully obtained, which can be confirmed by both HRTEM and XRD (**Figure** **2**A; Figure S1A,B, Supporting Information). Furthermore, the uniform dispersion of Cu can be confirmed through EDS mapping results, indicating the successful incorporation of Cu into HAT‐6CN (Figure 2A(c)). Additionally, the valence of Cu was also investigated by XPS. As illustrated in Figure 2C, two distinct peaks located at 933.6 and 953.5 eV can be observed, which can be ascribed to Cu^2+^ 2p_3/2_ and Cu^2+^ 2p_1/2_, respectively.^[^ ^19^ ^]^ Furthermore, to elucidate the binding configuration of HAT‐6CN and Cu within Cu HAT‐6CN, FTIR spectra before and after introducing Cu were further analyzed. As shown in Figure 2E, the nitrile stretching band for Cu HAT‐6CN was found at 2160 cm^−1^, indicating a lower energy level compared to the nitrile stretching band of free HAT‐6CN, which was observed at 2242 cm^−1^ (Figure 1D). Therefore, it is more likely that the copper ions coordinate with HAT‐6CN through the bipyridine sites rather than the terminal nitrile N‐binding sites (Figure S3, Supporting Information), which is expected to show two types of bands for nitrile stretching.^[^ ^20^, ^21^ ^]^ Deeper analysis was conducted with the zeta potential testing (Figure 2F). After ES adsorption, the zeta potential of Cu HAT rose from −11.4 to −9.7 mV, indicating electrostatic attraction between the Cu HAT and ES, which may be attributed to the self‐assembly of Cu HAT‐ES.^[^ ^22^ ^]^ Zeta potential of Cu HAT‐2DG was −11.5 mV due to the presence of a large quantity of ‐OH groups.^[^ ^23^ ^]^ In contrast, Zeta potential of Cu HAT‐2DG‐ES rose to −8.1 mV because ES was successfully loaded into Cu HAT‐2DG. Additionally, the Cu/N molar ratio in Cu HAT‐6CN was determined to be ≈4.0% through both ICP and elemental analysis. This observation signifies that a single HAT‐6CN molecule coordinates with three Cu ions.

### 2.3 Cell Uptake and Subcellular Localization Evaluation

**Figure 3:**
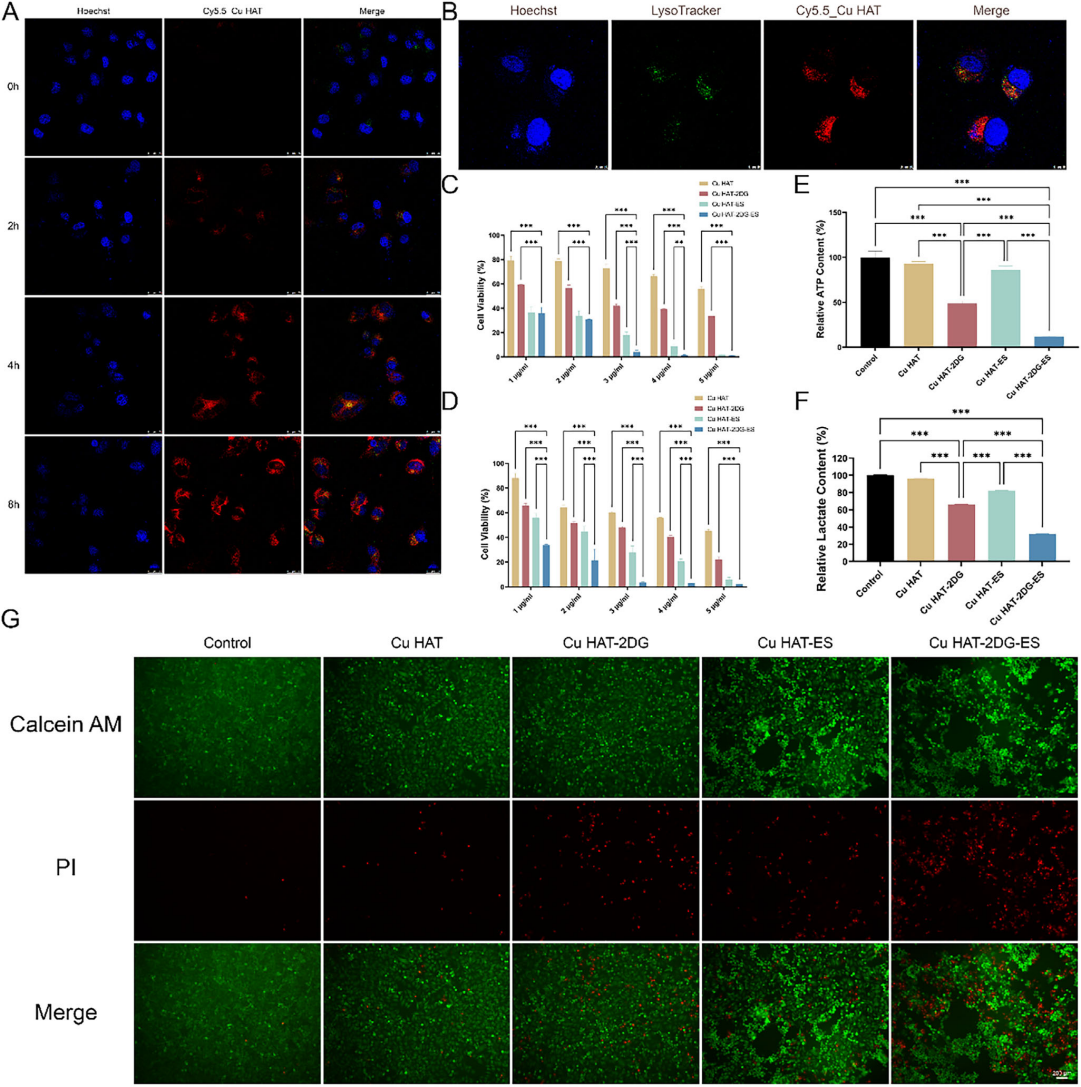
A,B) Cellular uptake of cy5.5‐labeled Cu HAT. C,D) Cell viability of Hepa1‐6 and Hep3b cells treated by different NTs for 24 h. E) Relative ATP and (F lactate content of Hep3b cells after being treated with different NTs for 24 h. G) Calcein AM and PI double staining results of Hep3b cells after incubation with different NTs for 12 h. (*n* = 5, *** indicates p < 0.001, ** indicates p < 0.01, * indicates p < 0.05).

Confocal laser scanning microscope (CLSM) was employed to detect the intracellular uptake and subcellular localization of Cu HAT. As shown in **Figure** **3**A,B, the accumulation of cy5.5‐labeled Cu HAT represents a time‐dependent manner in Hep3b cells and exhibited significant red fluorescence after 8 h. NTs mainly enter cells through cytosis, thereby incorporating the lysosome to be processed. Here, we used Lysotracker to characterize lysosomes and discovered the co‐localization of Cu HAT and lysosomes. Interestingly, most Cu HAT were released from lysosomes and entered the cytoplasm, promoting direct contact with molecules and its effect.

### 2.4 In Vitro Antitumor Efficiency

We evaluated the in vitro antitumor efficiency of the NTs against hepatic cancer cells via CCK‐8 assay and live/dead staining. As shown in Figure 3C,D, dose‐dependence cytotoxicity was revealed in all NTs, but a lower concentration of Cu HAT‐2DG‐ES was needed to induce complete in‐vitro tumor suppression in Hep3b and Hepa1‐6 cells. A consistent trend was also observed in H22 cells (Figure S4A, Supporting Information). Implementing 2DG and ES significantly increases the cytotoxicity of Cu HAT, indicating a synergistic killing effect.

The live‐dead staining assay exhibited a similar tendency to CCK‐8 (Figure 3G; Figure S4C, Supporting Information), where Cu HAT‐2DG‐ES leads to the most widespread cellular damage, verifying the intense antitumor capability of the NTs.

### 2.5 Starvation Therapy

Intracellular ATP and extracellular lactic acid were evaluated to confirm the effect of starvation therapy. As shown in Figure 3E,F, the intracellular ATP levels were effectively reduced by Cu HAT‐2DG, suggesting the inhibition of glycolysis caused by 2DG. However, tumor cells can utilize the TCA cycle for an alternative energy supply, limiting the effect of starvation therapy. Cuproptosis was found to disrupt the process of TCA cycle. Thus, we assumed and demonstrated that Cu HAT‐2DG‐ES could exacerbate energy deprivation in tumor cells by combining cuproptosis and starvation therapy. According to the results, ATP was sharply limited in the Cu HAT‐2DG‐ES group, as well as supernatant lactic acid.

### 2.6 Cuproptosis Detection

According to Tsvetkov et al., excessive intracellular copper could induce a novel form of regulated cell death named cuproptosis, presenting as loss of Fe‐S cluster proteins and aggregation of mitochondrial lipoylated proteins.^[^ ^24^ ^]^ Xu et al. demonstrated that GOx can facilitate cuproptosis in bladder cancer cells.^[^ ^25^ ^]^ Hence, our study assumed that Cu^2+^ ions released from Cu HAT will be transferred into and accumulate in the cytosome via ES, resulting in the CDT effect and cuproptosis. 2DG, a glucose analog for starvation therapy, was incorporated and supposed to sensitize cells to Cu HAT‐induced cuproptosis.

**Figure 4:**
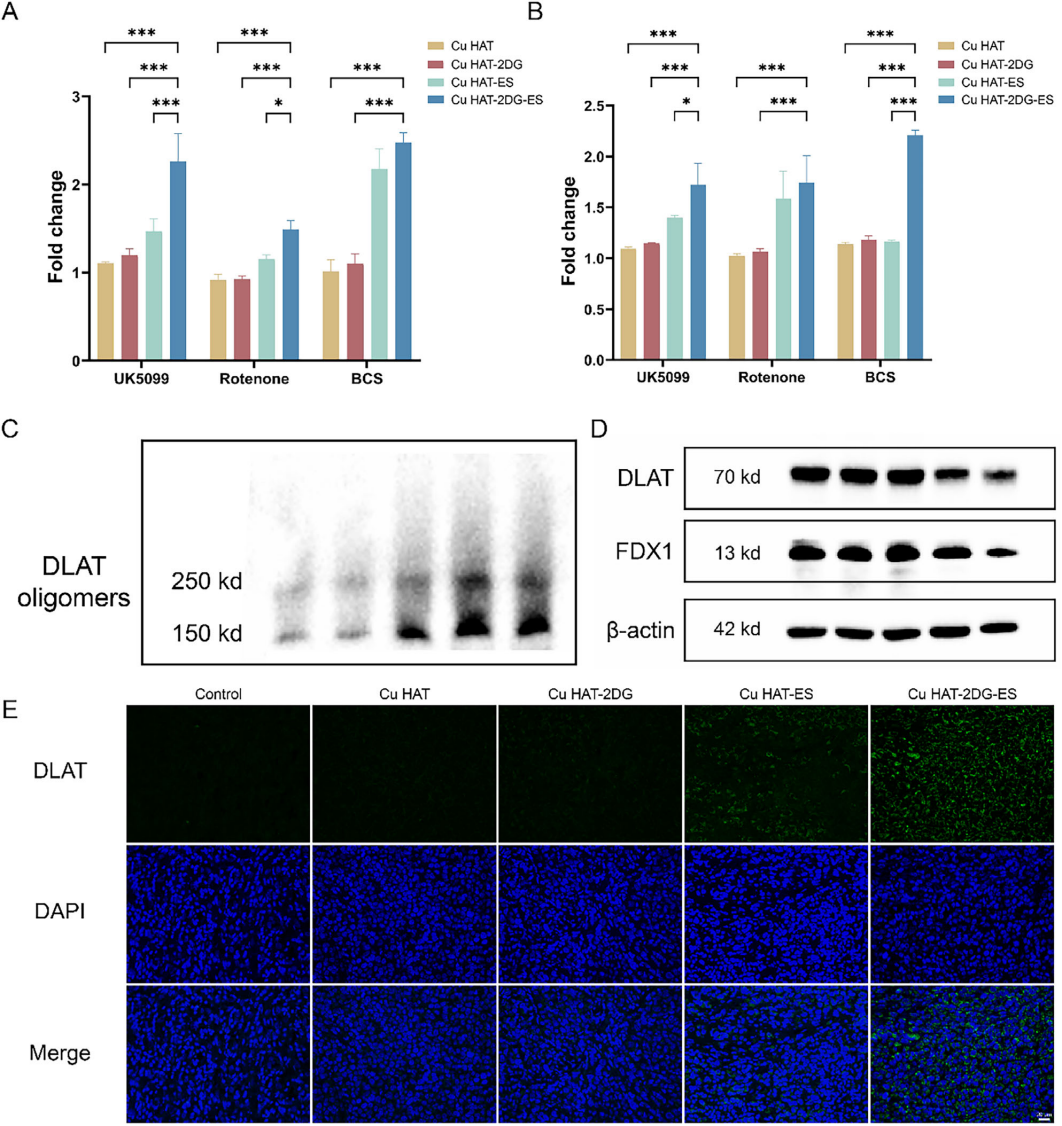
A) Relative cell viabilities of Hepa1‐6 and B) Hep3b cells treated by NTs in the presence of UK5099, Rotenone or BCS. C) DLAT oligomers and D) DLAT and FDX1 expression after 24 h incubation of PBS, Cu HAT, Cu HAT‐2DG, Cu HAT‐ES or Cu HAT‐2DG‐ES. E) Representative immunofluorescence images of tumor treated with NTs. (*n* = 5, *** indicates p < 0.001, ** indicates p < 0.01, * indicates p < 0.05).

We employed 3 cuproptosis inhibitors, UK5099, Rotenone, and BCS, to demonstrate the induction of cuproptosis by NTs. As shown in **Figure** **4**A,B, UK5099 has little effect on cell viability in cells treated with Cu HAT and Cu HAT‐2DG. However, it drastically rescued cell viability in Cu HAT‐2DG‐ES compared with other groups. The results of Rotenone and BCS present similar trends to UK5099, which indicated that cuproptosis plays a significant role in Cu HAT‐2DG‐ES‐induced cell death. Besides, 2DG and ES remarkably promoted this process.

Furthermore, we detected aggregation of DLAT proteins via non‐denaturing gel electrophoresis. Treatment with Cu HAT‐2DG‐ES leads to an obvious oligomerization of DLAT, while other therapies induced vague oligomerization, suggesting that cuproptosis was enhanced by CDT and starvation therapy (Figure 4C). Consistently, DLAT expression was found to decrease in cells treated with Cu HAT‐2DG‐ES. The expression of FDX1, a Fe‐S cluster protein, was also reduced (Figure 4D). We performed immunofluorescence on tumor specimens after in‐vivo treatment and found that, Cu HAT‐2DG‐ES induced the most significant DLAT foci, indicating the most significant DLAT aggregation in tumor (Figure 4E). As shown in Figure 1J, overexpressed DLAT is associated with reduced survival rates in HCC patients. Thus, Cu HAT‐2DG‐ES might improve HCC patient survival.

### 2.7 ROS Generation and GSH Depletion

**Figure 5:**
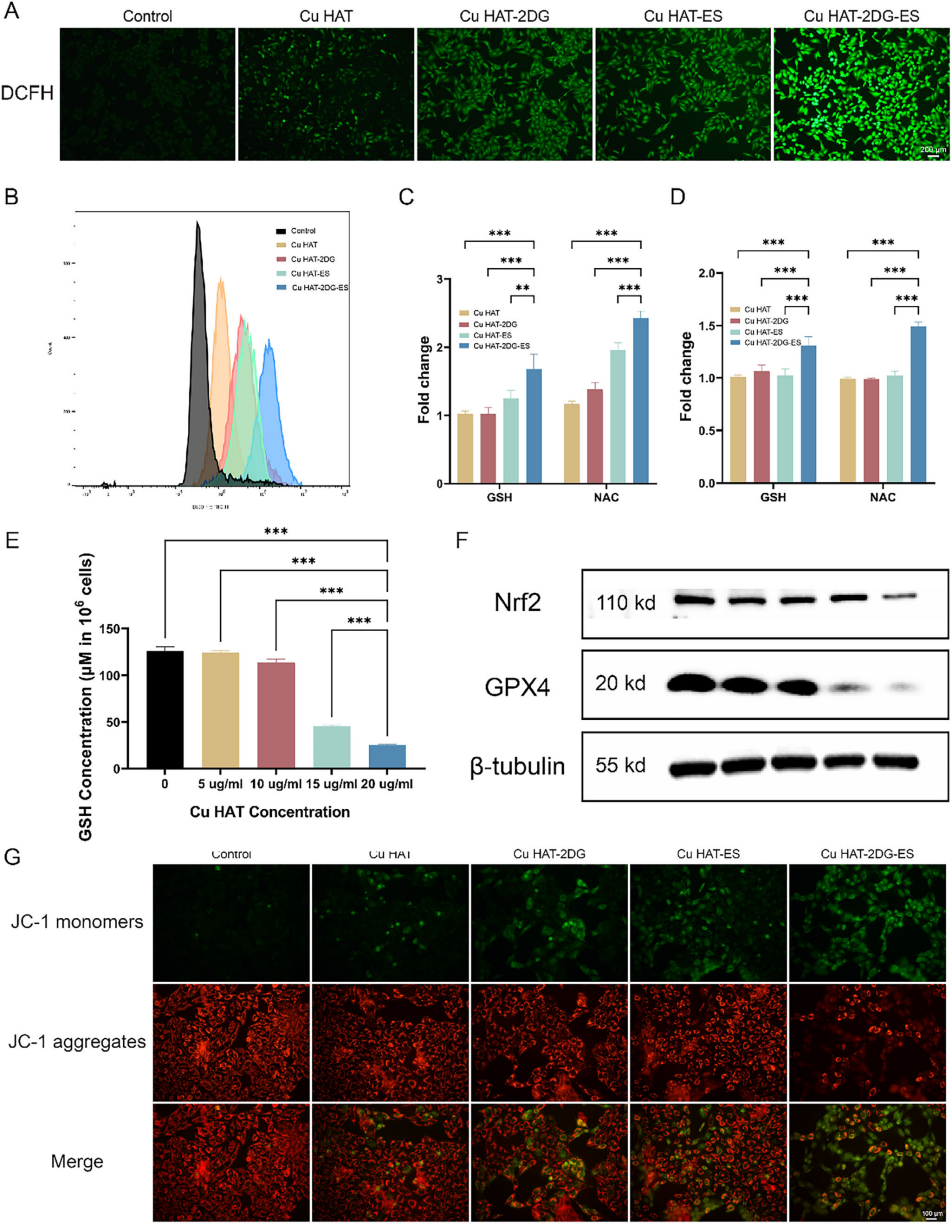
A) ROS of Hep3b cells detected with DCFH‐DA by fluorescence microscope and B) flow cytometry after treatment of different NTs for 6 h. C,D) Relative cell viabilities of Hepa1‐6 and Hep3b cells treated by NTs in the presence of GSH or NAC. E) Relative GSH concentration of Hep3b cells being treated with Cu HAT. F) Nrf2 and GPX4 expression after 24 h incubation of PBS, Cu HAT, Cu HAT‐2DG, Cu HAT‐ES or Cu HAT‐2DG‐ES. G) Mitochondrial membrane potential detection of Hep3b cells treated with NTs for 12 h using JC‐1 as probe. (*n* = 5, *** indicates p < 0.001, ** indicates p < 0.01, * indicates p < 0.05).

Next, we investigated the CDT effect of the NTs by detecting ROS generation and GSH depletion. DCFH‐DA probe (green fluorescence) was used to characterize intracellular ROS. Cu HAT‐2DG‐ES triggered the most remarkable elevation of ROS level among the treatments, reflected by the highest fluorescence intensity (**Figure** **5**A; Figure S4D, Supporting Information). In comparison, Cu HAT‐2DG and Cu HAT+Ele result in a moderate elevation of ROS. Then, we carried out flow cytometry (FCM) to confirm this effect. FCM revealed a quantified ROS level change among the groups, consistent with the fluorescence microscopic result (Figure 5B). Additionally, we found that Cu HAT‐2DG‐ES reduced the expression of NRF2 (Figure 5F), which is protective against excessive ROS. This might lead to further redox disturbances and intensify cellular damage.

Intracellular ROS and GSH level was found to be highly related to cytotoxicity. The cell viability increased significantly after the addition of GSH and NAC, especially in the Cu HAT‐2DG‐ES group, indicating the synergetic CDT effect (Figure 5C,D).

Subsequently, we determined the GSH level via the Glutathione Assay Kit due to Cu^2+^ being found to oxidize GSH into GSSG and result in a deteriorating redox balance. As shown in Figure 5E, Cu HAT caused a decrease in GSH level in a dose‐dependent manner. The depletion of GSH would affect the activity of GPX4, a protein that requires GSH as a cofactor. Thus, we conducted western blotting and demonstrated the inactivation of GPX4 (Figure 5F).

As shown in Figures 5G and S4B (Supporting Information), Cu HAT‐2DG‐ES significantly increased JC‐1 monomers, suggesting a decrease in mitochondrial membrane potential and mitochondrial damage.

### 2.8 Lipoic Acid Modification and Cuprotosis‐Related Stress Marker Assay

**Figure 6:**
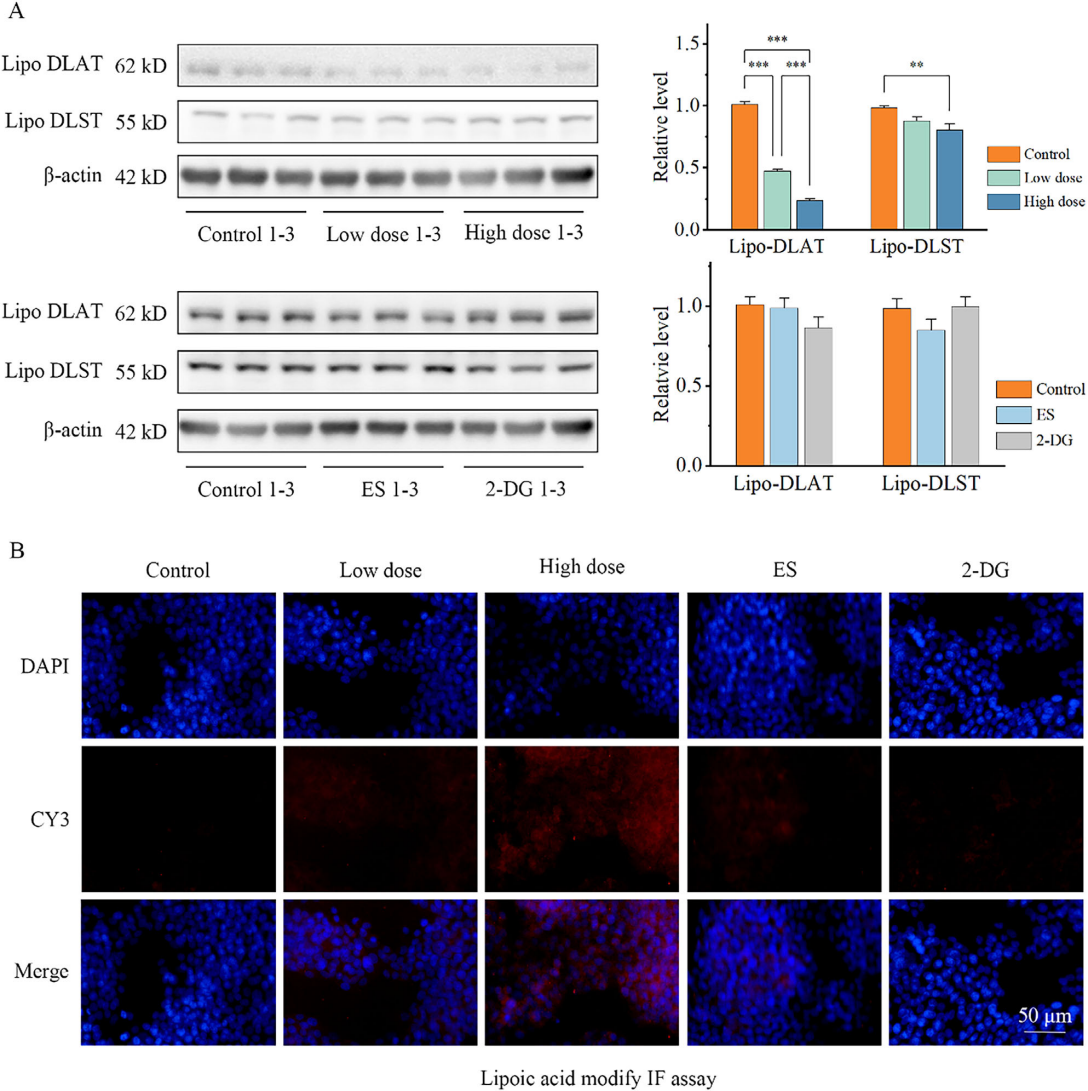
A) Lipoylation of Hep3b cells detected with Anti‐Lipoic acid primary antibody by Western‐blot and B) immunofluorescence after treatment of ES, 2DG and different dose of NTs for 24 h. (*n* = 5, *** indicates p < 0.001, ** indicates p < 0.01, * indicates p < 0.05).

Then, we investigated the lipoic acid modification effect of the NTs by detecting lipoic DLAT and lipoic DLST expression as a representative. Lipoic acid primary antibody was used to characterize the expression of lipoic DLAT/DLST by western‐blot. Cu HAT‐2DG depleted lipoic DLAT expression level as expected in a dose‐dependent manner. ES also deleted lipoic DLAT expression while 2DG showed slight effect on lipoic DLAT expression. Also Cu HAT‐2DG triggered the most remarkable down‐regulation of lipoic DLAT level among the treatments in IF assay, reflected by fluorescence intensity (**Figure** **6**A,B).

**Figure 7:**
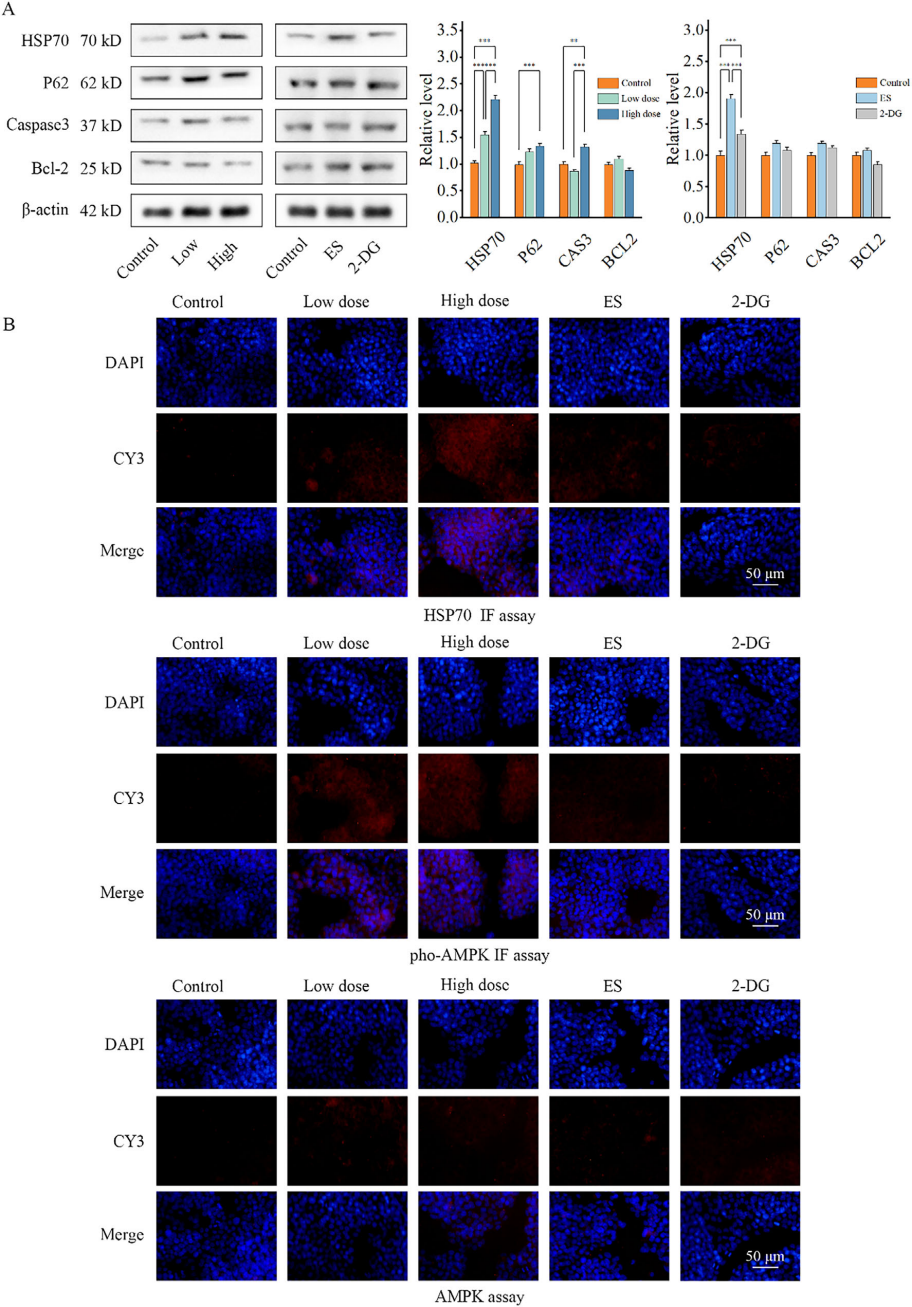
A) Cell stress markers related to cuprotosis of Hep3b cells detected with Anti HSP70, P62, Caspase3, Bcl‐2 primary antibody by Western‐blot and B) with Anti HSP70, AMPK, pho‐AMPK(Thr172) primary antibody by immunofluorescence after treatment of ES, 2DG and different dose of NTs for 24 h. (*n* = 5, *** indicates p < 0.001, ** indicates p < 0.01, * indicates p < 0.05).

Then, we carried out cuprotosis‐related stress marker detection to confirm the cuprotosis‐related stress effect. Western‐blot and IF revealed quantified level changes in HSP70, P62, Caspase3, Bcl‐2 among the NT dose‐dependent groups, consistent with the fluorescence microscopic result (**Figure** **7**A,B). However, in ES and 2DG groups the expression level revealed slight change. This may indicate a more efficient effect of Cu HAT in cuprotosis and stress induction than that in ES.

Lipoic modification level was found to be highly related to cuprotosis. Lipoic modification level of DLAT and DLST were regarded as key markers of cuprotosis. The Lipoic modification decreased significantly after the addition of CuHAT and ES groups, especially in the Cu HAT‐2DG‐ES group. And at the same time, Cu HAT‐2D‐ES group indicated obvious cell stress induction while 2DG and ES groups showed slight cell stress induction (Figure 6).

Subsequently, we determined that Cu HAT could promote a complex induction of cuprotosis and cell stress. This complex induction leads to cytotoxic protein stress and then cell death.

### 2.9 In Vivo Antitumor Effects

**Figure 8:**
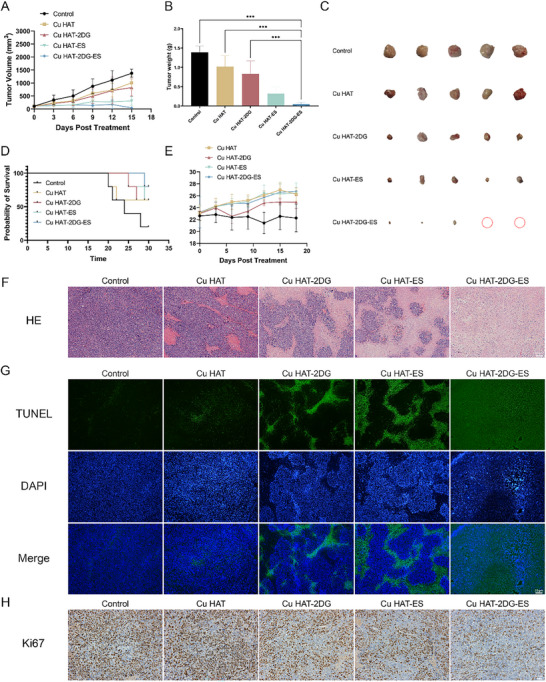
A) Tumor growth curves and B) tumor weight and C) tumor images of mice with various treatments. D) Mice survival rate within 30 days. E) Bodyweight change curves of all groups. F) The representative H&E images of tumor slides after different treatments. G) Immunofluorescence staining of DLAT of the tumor tissues after different treatments. H) Immunochemistry staining of Ki67 of the tumor tissues after different treatments. (*n* = 5, *** indicates p < 0.001, ** indicates p < 0.01, * indicates p < 0.05).

Next, we established a subcutaneous tumor model in C57 mice to confirm the in vivo antitumor efficacy of NTs (*n* = 5). Mice were injected intravenously with i) PBS, ii) Cu HAT, iii) Cu HAT‐2DG, iv) Cu HAT‐ES, v) Cu HAT‐2DG‐ES at a dosage of 10 mg kg^−1^. As shown in **Figure** **8**A–C, tumor in the PBS group reached the most significant volume, while Cu HAT inhibited tumour growth slightly. Cu HAT‐2DG and Cu HAT‐ES induced a certain but not complete tumor growth inhibition, indicating that starvation or CDT therapy alone was insufficient for tumor suppression. Notably, tumor growth was significantly inhibited after Cu HAT‐2DG‐ES treatment, suggesting the efficient synergistic effect of cuproptosis, starvation therapy and CDT. In survival analysis, the lifespan of mice in the Cu HAT‐2DG‐ES group was significantly prolonged compared to other groups (Figure 8D).

No significant body weight fluctuations occurred during treatment Figure 8E. Stained images of major organs’ hematoxylin & eosin (H&E) and blood biochemistry also revealed negligible changes (Figures S5 and S6, Supporting Information). Additionally, we conducted H&E staining and terminal deoxynucleotidyl transferase dUTP nick end labelling (TUNEL) assay to evaluate the morphologic changes in tumor tissue. As shown in Figure 8F,G, Cu HAT‐2DG‐ES treatment led to the most prominent necrosis and apoptosis. To further investigate the mechanism of therapeutic effects, immunohistochemical assays of Ki‐67 of tumor specimens were also conducted (Figure 8H). Cu HAT‐2DG‐ES downregulated Ki‐67 expression, confirming that the synergetic effect can restrain tumor proliferation.

**Figure 9:**
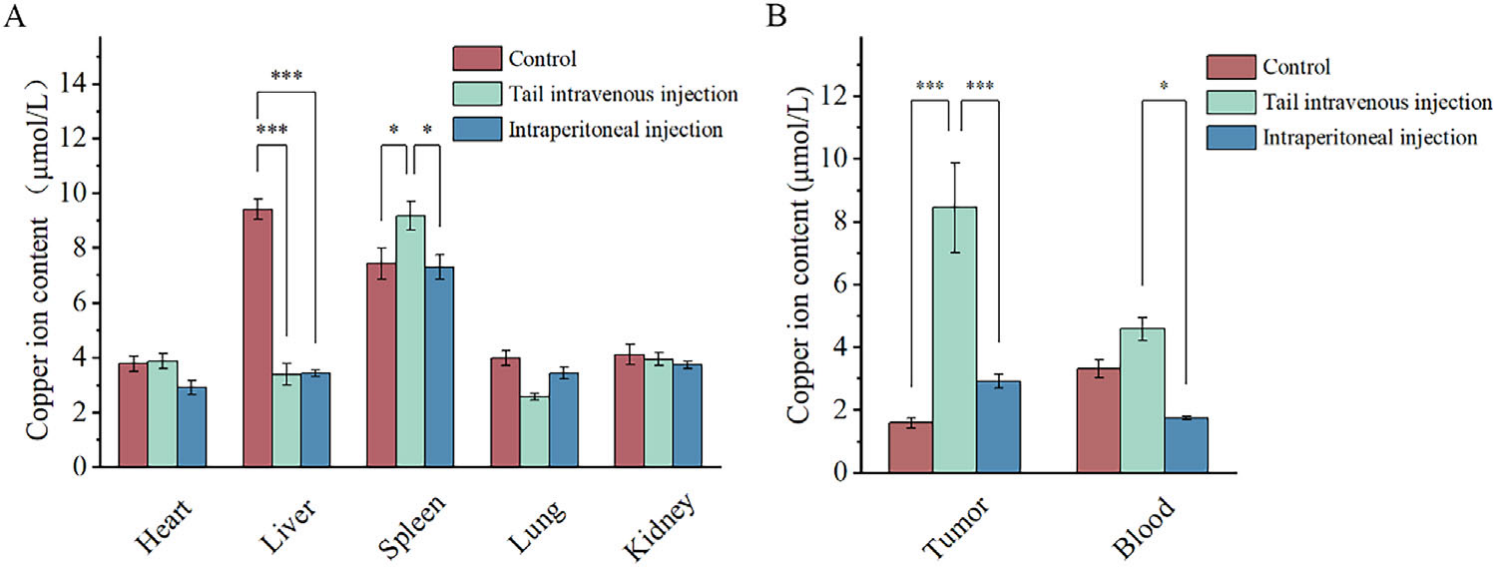
Copper ion content in main tumor and main organs of H22 tumor‐bearing murine models tested by ion complexometry after treatment with different method of NTs after five‐time injection in total. A) Copper ion content in different organs. B) Copper ion content in main tumor and peripheral blood. (*n* = 5, *** indicates p < 0.001, ** indicates p < 0.01, * indicates p < 0.05).

Then we detected copper ion content in a tumor bearing murine model. After three times injection of NTs in different methods, we tested copper ion content with complexation precipitation method. In the test, we found that the tumor‐site copper content in tail intravenous injection group was higher than that in intraperitoneal injection group. This phenomenon indicated that NT's comparative tumor targeting through blood route. Even though copper's metabolism in the liver was consensus, the test did not show obvious copper accumulation in the liver. This phenomenon may indicate NT's selective accumulation or the characteristic of being difficult to accumulate in the short term. NT's selective accumulation may contribute to fewer side effects in clinical usage (**Figure** **9**).

## 3 Conclusion

We identified cuproptosis as a potential modality for HCC treatment and synthesized a copper‐based nanoplatform combining copper ionophores and starvation therapy, which was confirmed to have a synergistic therapeutic effect on HCC in both in vitro and in vivo experiments. This study provides an alternative approach for clinical HCC treatment and provides an enhancement method for copper ionophores in tumor treatment.

## 4 Experimental Section

### Cell Culture

Hepa1‐6, Hep3b, and H22 cell lines were obtained from Procell Life Science and Technology. Hepa1‐6 and Hep3b cells were cultured in Dulbecco's Modified Eagle's Medium (DMEM) containing 10% fetal bovine serum and 1% penicillin‐streptomycin solution. H22 cells were cultured in Roswell Park Memorial Institute 1640 medium (RPMI 1640) containing 10% fetal bovine serum and 1% penicillin‐streptomycin solution. All cells were incubated under 37 °C and 5% CO_2_ condition.

### GSH Assay

Glutathione Assay Kit (Beyotime Biotechnology, China) was used to determine the intracellular GSH content. Hep3b cells were co‐incubated with 5–20 µg mL^−1^ Cu HAT for 24 h and were harvested for GSH assay following the manufacturer's instruction.

### ATP Assay

To measure the intracellular ATP concentration, ATP Assay Kit (Beyotime Biotechnology, China) was used after co‐incubation with 3 µg mL^−1^ NTs. Briefly, Hep3b cells were seeded in 10 mm dishes overnight. Then, NTs were added and co‐incubated for 24 h, and cells were harvested for ATP assay following the manufacturer's instruction.

### Lactate Assay

Lactic Acid assay kit (Nanjing Jiancheng Bioengineering Institute) was employed to detect the extracellular lactic acid concentration following the manufacturer's instruction. Briefly, Hep3b cells were seeded in 10 mm dishes overnight and then co‐incubated with 3 µg mL^−1^ NTs for 24 h. Then the supernatant was collected for lactic acid assay.

### Intracellular ROS Detection

DCFH‐DA (Dojindo Laboratories, Japan) was used to measure the intracellular ROS level. Briefly, Hep3b and Hepa1‐6 cells were seeded in 12‐well plates (1*10^5^ per well) and incubated for 12 h. Then, the medium was replaced by fresh medium containing 2 µg mL^−1^ NTs. After 6 h of co‐incubation, the medium was discarded and washed with PBS twice. DCFH‐DA probe was added under the manufacturer's instruction and incubated for 30 min. Then, the cells were washed with PBS and observed by fluorescence microscope.

### Live‐Dead Cell Staining Assay

Calcein‐AM and propidium iodide (Beyotime Biotechnology, China) were used for in vitro live‐dead detection. Briefly, Hepa1‐6 and Hep3b cells were seeded in 12‐well plates (1*10^5^ per well) overnight and followed by co‐incubation with 3 µg mL^−1^ NTs for 12 h. Cells were stained with Calcein‐AM and propidium iodide for 30 min and then imaged by fluorescence microscope.

### Cell Viability Assay

Cell counting kit‐8 (Abmole, China) was used for cell viability measurement. Hep3b, Hepa1‐6 and H22 cells were seeded in 96‐well plates overnight and co‐incubated with 1–5 µg mL^−1^ NTs for 24 hours. The medium was replaced by fresh medium containing 10% CCK8 for 40–60 min. Finally, the absorbance value at 450 nm was detected by the microplate reader.

### Western Blotting Assay

Nrf2, GPX4, DLAT, FDX1, Lipoic acid, HSP70, P62, Caspase3, Bcl‐2 expression were determined by western blotting. Briefly, Hep3b cells were seeded in 10 mm dishes and treated with 3 or 5 µg mL^−1^ NTs for 24 h. Then, the total protein was extracted by lysis buffer and subjected to standard SDS‐polyacrylamide gel electrophoresis (SDS‐PAGE) and LDS‐PAGE followed by immunoblotting. Oligomerization of DLAT was detected by nondenaturing gel electrophoresis. After incubation with primary antibody and horseradish peroxidase (HRP)‐conjugated secondary antibody, the protein expression was visualized by the ECL detection system.

### In Vivo Experiment

Male C57BL/6 mice (4‐6 weeks old) were purchased from and fed in Experimental Animal Center of Xi'an Jiaotong University. All animal procedures were approved by Institutional Animal Care and Use Committee of Xi'an Jiaotong University (Number. XJTUAE2023‐231).

H22 cells (2*10^6^ per mouse) were subcutaneously injected into the right thigh of each mouse. The mice were randomly assigned to five groups: i) PBS, ii) Cu HAT, iii) Cu HAT‐2DG, iv) Cu HAT‐ES, v) Cu HAT‐2DG‐ES) and received treatments until the tumor volume reached 100 mm^3^. 10 mg kg^−1^ of PBS or NTs were administrated intravenously for each mouse and treated every 3 days. The tumor volume and body weight were recorded every three days. The tumor volume was calculated as width^2^*length/2. The main organ and tumor were harvested on day 15.

H22 cells (2*10^6^ per mouse) were subcutaneously injected into the right infracostal area of each mouse. The mice were randomly assigned into three groups: i) Control (Saline), ii) Cu HAT tail intravenous injection group, iii) Cu HAT intraperitoneal injection group and received treatments of 100 µL saline or 100 µL CuHAT (10 mg kg^−1^) every two days. The main organ, blood and tumor samples were collected after three‐time treatments. The solid samples were weighed and homogenized on ice by ultrasonic cell crusher in saline and blood samples were collected in heparin anticoagulant tubes. Then copper ion content was detected according to the manufacturer's manual with copper ion detection kit (Nanjing Jiancheng Bioengineering Institute, Nanjing, China).

### Statistical Analysis

Prior to analysis, dataset normality was assessed using the Shapiro‐Wilk test and potential outliers were systematically evaluated through residual analysis. Continuous variables were presented as mean ± standard deviation (SD). Intergroup comparisons were performed using one‐way analysis of variance (ANOVA) with subsequent two‐tailed Dunnett's post‐hoc testing for multiple comparisons against a specified control group. The family‐wise error rate was controlled through Bonferroni correction at α = 0.05. Sample sizes (n) for individual experiments were detailed in respective figure captions. All statistical procedures, conducted using SPSS 18.0 (SPSS Inc., USA), maintained a significance threshold of p < 0.05 after multiplicity adjustment.

## Conflict of Interest

The authors declare no conflict of interest.

## Supporting information



Supporting Information

## Data Availability

Research data are not shared.
