# Supplementary material for: Copper‐Based Nanotubes That Enhance Starvation Therapy Through Cuproptosis for Synergistic Cancer Treatment
Source: Adv Sci (Weinh). 2025 Jun 5;12(32):e04121. doi: 10.1002/advs.202504121 (PMC12407334; doi:10.1002/advs.202504121)
Supplement: Supplementary file 1 — Supporting Information [file ADVS-12-e04121-s001.docx]

**Supplementary Materials**

Copper-based Nanotubes that enhance Starvation Therapy through Cuproptosis for Synergistic Cancer Treatment

Xuan Han^a,b,1^, Xin Zhang^c,1^, Zhiping Liu^d^, Hang Liu^a,b^, Derek Wu^e^, Yinli He^f^, Kexin Yuan^b^, Yi Lyu^a,b,d^*, Xiaofei Liu^a,b,d^*

^a^ Department of Hepatobiliary Surgery, The First Affiliated Hospital of Xi’an Jiaotong University, Xi’an, Shaanxi Province, China

^b^ Center for Regenerative and Reconstructive Medicine, Med-X Institute, The First Affiliated Hospital of Xi’an Jiaotong University, Xi’an, Shaanxi Province, China

^c^ Department of Orthopedics, The Second Affiliated hospital of Air Force Medical University (AFMU), Xi’an, Shaanxi Province, China

^d^ National Local Joint Engineering Research Center for Precision Surgery & Regenerative Medicine, Shaanxi Provincial Center for Regenerative Medicine and Surgical Engineering, The First Affiliated Hospital of Xi’an Jiaotong University, Xi’an, Shaanxi Province, China

^e^ Brooklyn College, the City University of New York, Brooklyn, NY, United States

^f^ BioBank, The First Affiliated Hospital of Xi'an Jiaotong University, Xi'an, Shaanxi Province, China

*Correspondence: luyi169@126.com; [xiaofeiliu@xjtu.edu.cn](mailto:xiaofeiliu@xjtu.edu.cn)

^1^These authors contributed equally to this work and should be considered co-first authors

**Chemicals**

1,4,5,8,9,12-hexaazatriphenylene hexacarbonitrile (HAT-6CN) was purchased from Shanghai Tensus Biotech Co., Ltd. Copper(II) perchlorate was obtained from J&K Scientific Ltd. All chemicals were used without further purification. 1,2-distearoyl-sn-glycero-3-phosphoethanolamine-N-[methoxy(polyethylene glycol)-750 (DSPE-PEG2000), ES, 2DG was purchased from Xi’ an Ruixi Biological Technology Co,. Ltd.

Synthesis of Cu HAT, Cu HAT-2DG, Cu HAT-ES, Cu HAT-2DG-ES

To synthesize Cu HAT, 0.5 mmol of copper(II) perchlorate was dissolved in 20 mL of water, while 0.2 mmol of 1,4,5,8,9,12-hexaazatriphenylene hexacarbonitrile (HAT-6CN) was dissolved in 20 mL of acetonitrile. Upon combining these solutions, a crystalline precipitate formed after evaporating at 50 °C; this is Cu HAT.

To synthesize Cu HAT-ES, 10 mg of Cu HAT, 1 mg ES, 1 mg DSPE-PEG2000 was dissolved in 5 mL of iced water in ultrasound for 3 h. Afterwards, these samples were freeze-dried.

To synthesize Cu HAT-2DG, the above procedure was repeated using 2DG instead of ES.

To synthesize Cu HAT-ES-2DG, the above procedure was repeated using both 2DG and ES.

**Characterization**

X-ray diffraction (XRD) patterns were collected using an X-ray diffractometer (Bruker D8 ADVANCE diffractometer). High-resolution transmission electron microscopy (HRTEM) was conducted using the JEM-F200 instrument. X-ray photoelectron spectroscopy (XPS) measurements were conducted using the Thermo Fisher ESCALAB Xi+ spectrometer. The Cu content was analyzed using ICP-OES with the NexION 350D instrument. The N content was confirmed by element analysis (EuroEA3000). The Fourier-transform infrared spectroscopy (FT-IR) spectrum was acquired using the VERTEX70 FT-IR spectrometer.


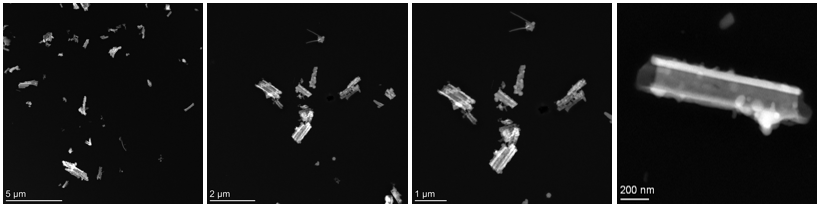


Fig.S1 High-resolution TEM images of Cu-HAT-6CN.


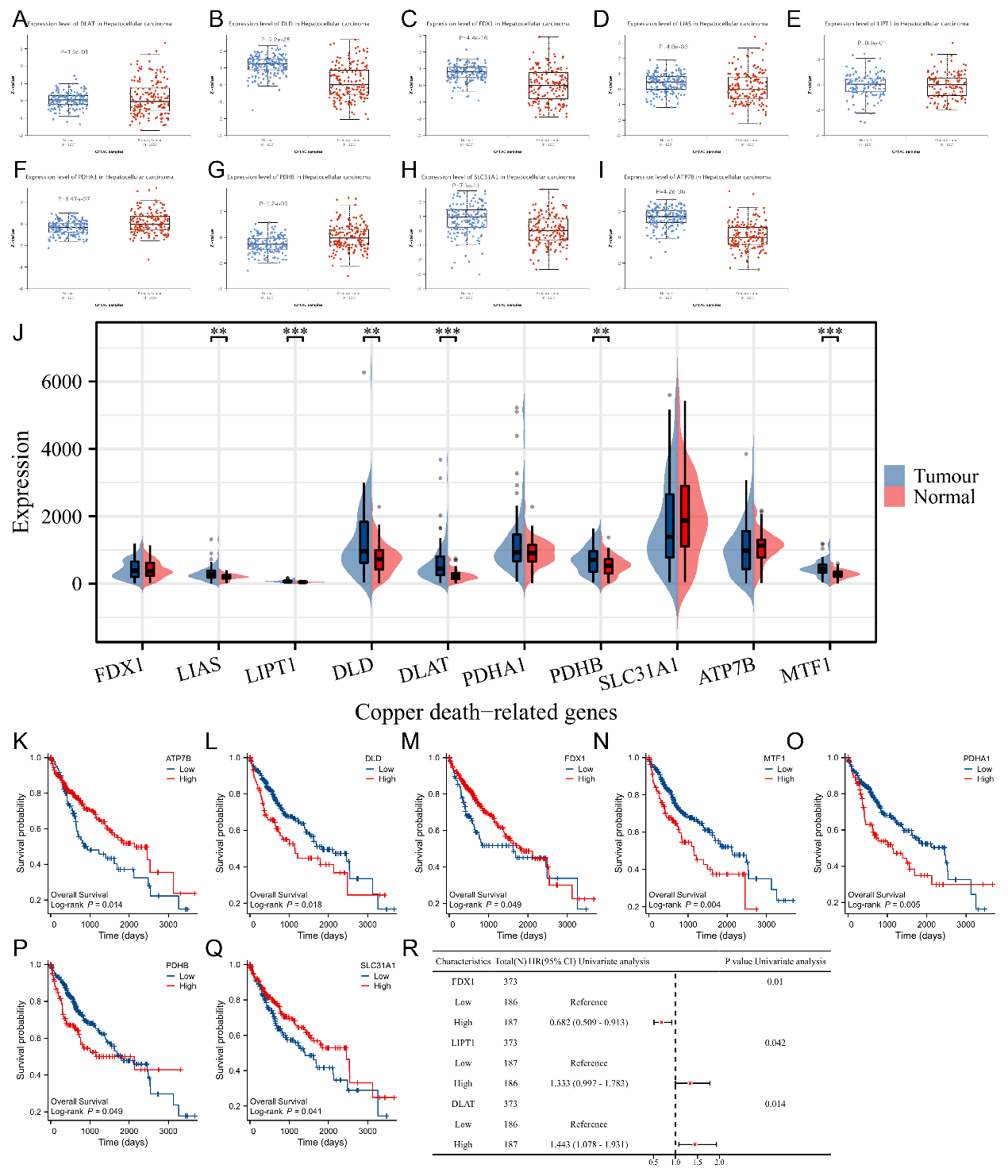


Fig.S2 A-I) Expression of cuproptosis-related genes in CPTAC database. J) Expression of cuproptosis-related genes in GEO database. K-Q) Survival analysis of cuproptosis-related genes. R) Cox analysis of FDX1, LIPT1 and DLAT.


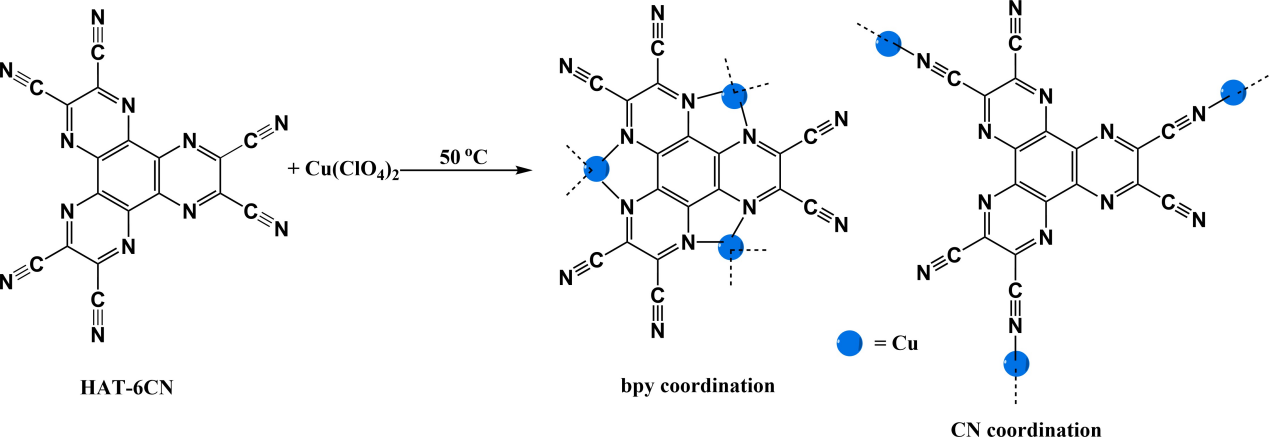


Fig.S3 Two possible modes of coordination of Cu in Cu-HAT-6CN.


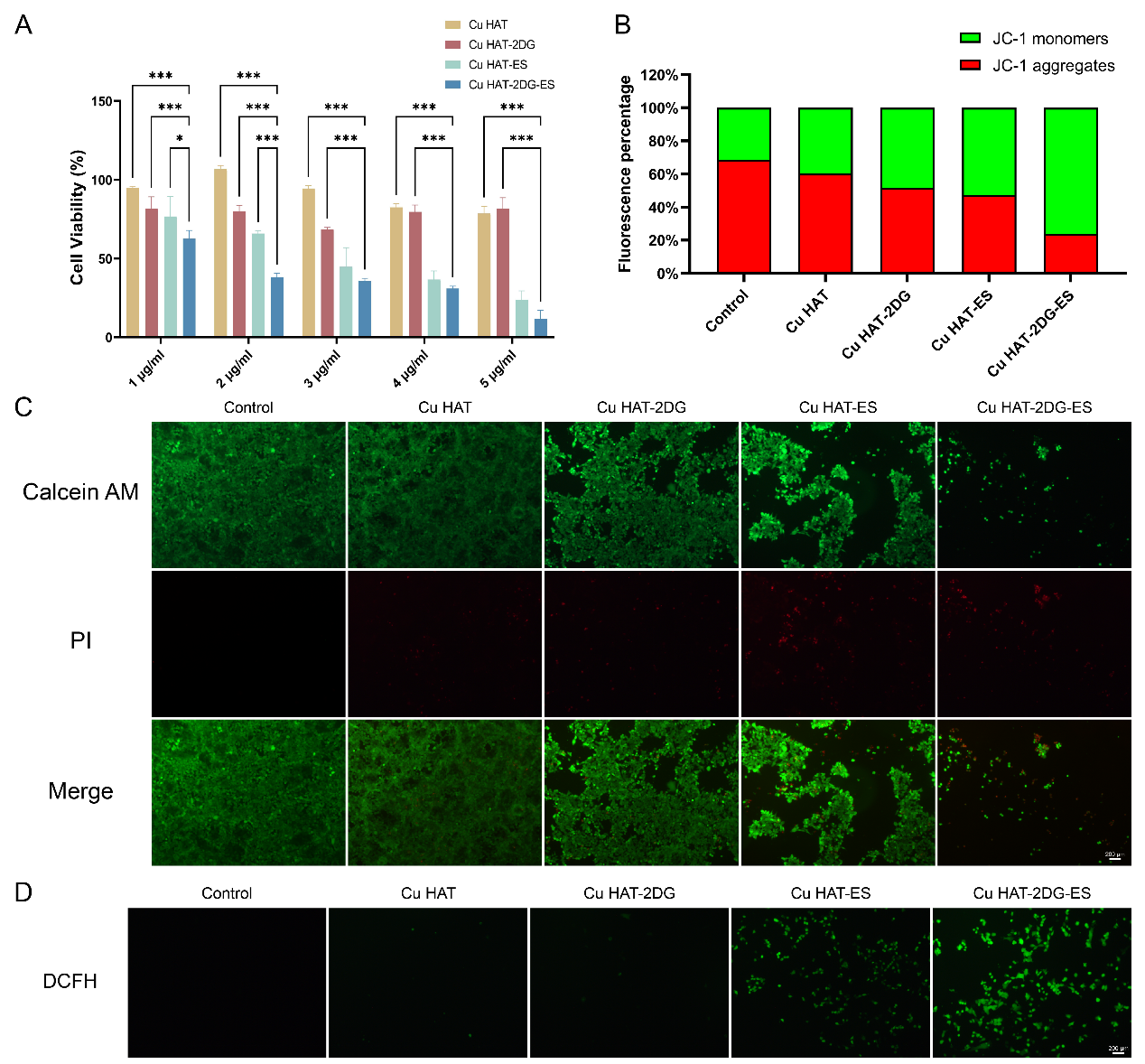


Fig.S4 A) Cell viability of H22 cells treated by different NTs for 24 hours. B) Quantification of JC-1 results. C) Calcein AM and PI double staining results of Hepa1-6 cells after incubation with different NTs for 12 hours. D) ROS of Hepa1-6 cells detected with DCFH-DA by fluorescence microscope. (n=5, *** indicates p<0.001, ** indicates p<0.01, * indicates p<0.05)


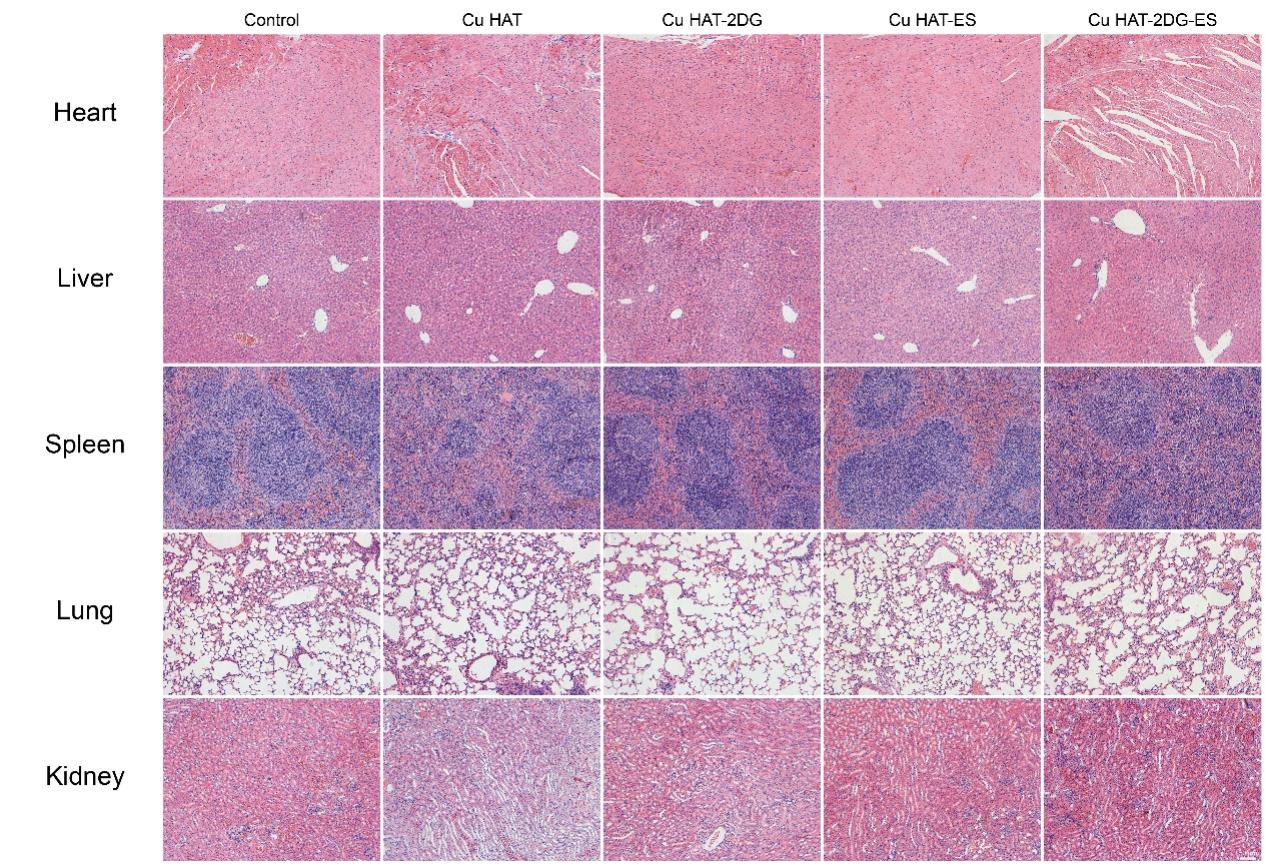


Fig.S5 H&E staining of major organs after different treatments.


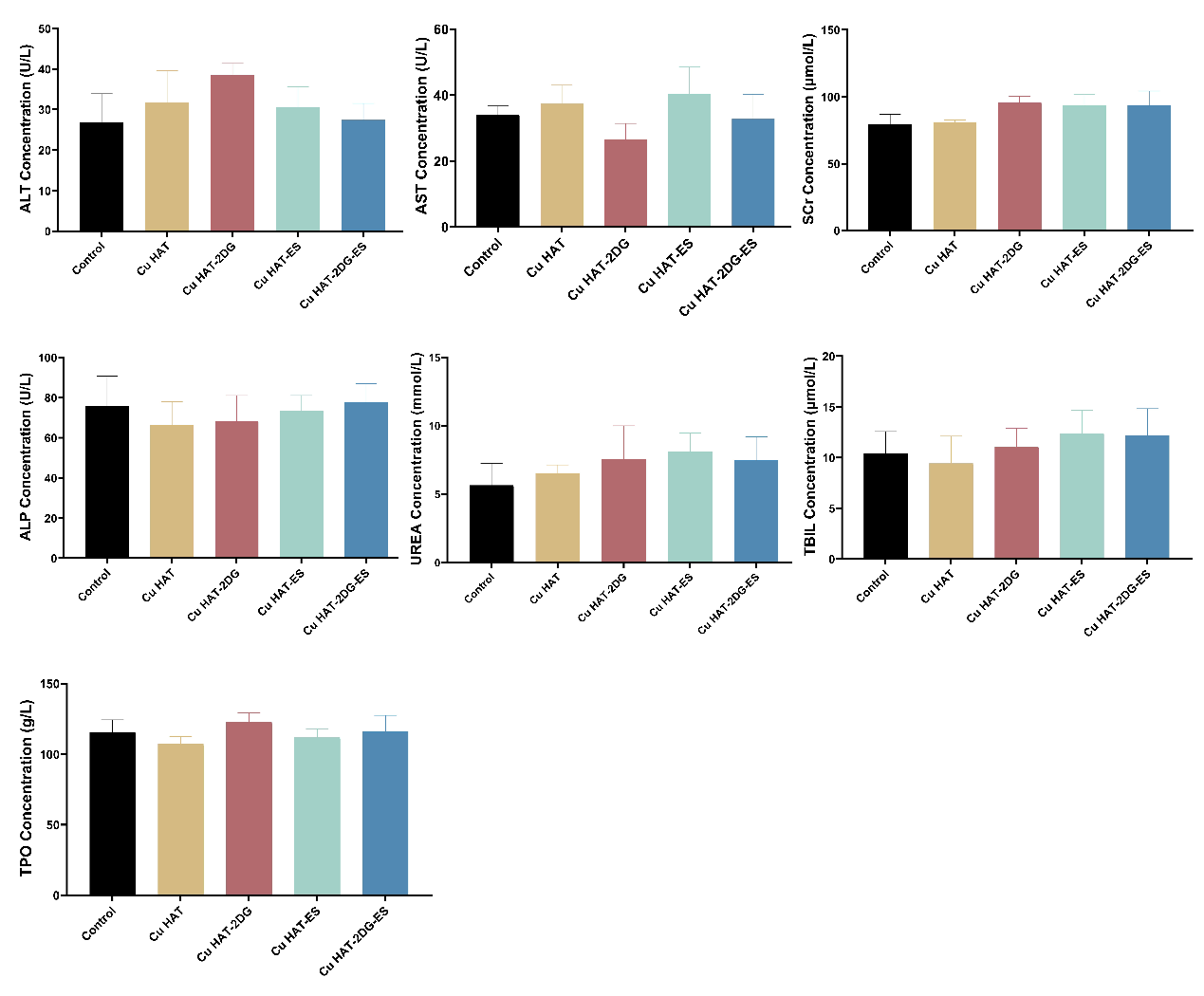


Fig.S6 The blood biochemical indicators after different treatments.
